# Prescriptions and Dispensing

**Published:** 1907-11-09

**Authors:** 


### Therapeutics.

### PRESCRIPTIONS AND DISPENSING.

### V. MEDICINES IN LIQUID FORM.

Mixtures (*continued*).

We were discussing in our last issue the errors of typical prescriptions, of which the first was the following:—

| | | |
|---|---|---|
| ℞ | Bismuthi Carbonatis ... ... ... | ʒiij. |
| | Tincturæ Cardamorum compositæ ... | ℥ss. |
| | Acidi Hydrocyanici diluti ... ... | ʒss. |
| | Aquam Chloroformi ... ... | ad ℥vj. |

In all the cases the remedy for the imperfections of the mixtures lies partly in increasing the viscosity of the vehicle by a suitable addition; in the first case it is further necessary to bring the bismuth carbonate into the finest possible powder; the minute particles, in a somewhat viscous medium, then settle with comparative slowness after thorough shaking. The other two cases require that the precipitate be produced in such a way that the particles of quinine salicylate and precipitated balsam respectively do not come into complete contact wth each other, so that they cannot coalesce into clots.

Many substances—sugar, glycerine, gum, and so forth—would increase the viscosity of the vehicle, but of all these gum is by far the most efficient; a smaller quantity of it than of the others suffices, and as it is itself inert and practically without any effect on the taste of the mixture, its addition is permissible where the addition of sugar, glycerin, or similar substances would not be. Two official gums are available for the purpose, namely, *acacia* and *tragacanth*. It is not a matter of indifference which is used, each being preferred in certain cases. When the principal requirement is to increase viscosity, as in the first two of the above examples, tragacanth is the more serviceable. Where the function of the gum is chiefly to coat the particles of a precipitate and keep them apart, as in the third example, acacia is the better.

Turning back to our discussion of the first of the above prescriptions, and assuming that it has been corrected by the addition of gum tragacanth, the first thing in dispensing it is to rub the bismuth carbonate up into a very fine powder in a mortar. The salt is always supplied in powder, but it is by no means equally fine, some samples containing a considerable proportion of dense particles which would fall to the bottom of any mixture far more rapidly than the fine fragments powdered by rubbing the salt well in a mortar. Some powdered tragacanth is next added to the powder, about 12 grains being sufficient for the 3 drachms of bismuth carbonate; the two are then mixed well in the dry state. The compound tincture of cardamoms is next added and triturated with the powders, and while still stirring, about 1 ounce of the chloroform water is added. After stirring until the contents of the mortar are homogeneous and smooth, the latter are diluted with further portions of chloroform water and transferred to the bottle; the mortar should be well rinsed with further quantities of the chloroform water, the bottles being corked and well shaken after transferring each addition to it. The hydrocyanic should be added last of all, for the reasons we have already given.

If the tincture is not poured on to the powders, but water added directly to them, it is difficult to prevent the tragacanth mucilage from being formed in lumps which it is almost impossible to break down afterwards; for this reason any alcoholic liquid ordered should always be added to tragacanth before bringing it into contact with water, a point which forms the basis for the official formula for making *mucilago tragacanthæ*, namely put 120 minims of 90 per cent. alcohol into a 20-ounce dry bottle, and add 60 grains of gum tragacanth in fine powder; shake till evenly moistened by the spirit, and then add distilled water to 10 ounces; shake again quickly to make a uniform mucilage.

Glycerin will do instead of spirit if it is an ingredient of the mixture prescribed.

If *bismuth subnitrate* is prescribed in place of the carbonate, a new difficulty arises; on mixing this salt with water, a small quantity of nitric acid is liberated, the salt becoming more basic, and this nitric acid causes gelatinisation of the tragacanth mucilage after a longer or shorter time. This acidity may be neutralised by adding a very little ammonia, and the compound tragacanth powder may then be used as the suspending agent. The best plan is to rub the bismuth subnitrate in a mortar and add about half the chloroform water in portions, transferring the mixture to the bottle and shaking well; then add to this three drops of the official liquor ammoniæ for each drachm of subnitrate, and triturate well; add this mixture gradually to the compound tragacanth powder in a mortar, taking 30 grains of the latter for each drachm of subnitrate, and triturate till smooth; transfer to the bottle, and rinse the mortar with further portions of the chloroform water. If any of the other ingredients of the required mixture makes the use of ammonia objectionable, the suspending must be done with gum acacia with about one-fifth of its weight of compound tragancanth powder, 30 and 6 grains respectively to each drachm of bismuth salt; in this case the subnitrate should be rubbed up with half the chloroform water, and the gums with the other half, and the two portions mixed in the bottle by shaking.

Whilst we are upon the subject of bismuth mixtures we may notice another point, which is raised by the following prescription:—

| | | |
|---|---|---|
| ℞ | Bismuthi Subnitratis ... ... ... | ʒij. |
| | Sodii Bicarbonatis ... ... ... | ʒij. |
| | Syrupi Aurantii ... ... ... ... | ℥ss. |
| | Tincturæ Genitanæ compositæ ... ... | ʒij. |
| | Aquam ... ... ... ... | ad ℥viij. |

Bismuth subnitrate and sodium bicarbonate mixed together with water slowly react, bismuth subcarbonate and sodium nitrate being formed, and half the carbonic acid of the bicarbonate being set free. This reaction proceeds so slowly that the mixture may seem a good one when it leaves the doctor's dispensary, but gradual evolution of $CO_2$ will continue, and the cork will be blown out of the bottle, or the latter may even burst, perhaps when it is in the patient's hands.

(*To be continued.*)

